# P59 Antimicrobial Allergy Week—an educational campaign at a large teaching hospital to improve knowledge of the impact of antimicrobial allergies

**DOI:** 10.1093/jacamr/dlag102.065

**Published:** 2026-06-26

**Authors:** Kelly Atack, Sarah Denman

**Affiliations:** Leeds Teaching Hospitals NHS Trust, Leeds, UK; Leeds Teaching Hospitals NHS Trust, Leeds, UK

## Abstract

**Background:**

National Penicillin Allergy Day is celebrated on 28 September each year and there is currently a significant focus on reduction of inaccurate penicillin allergies as an antimicrobial stewardship initiative as an inaccurate label can lead to an increased risk of healthcare associated infections, patient harm due to side effects of second line antimicrobials and mortality. In Leeds Teaching Hospitals NHS Trust, we have a penicillin allergy assessment and management guideline and aimed to use the week to promote this guidance across the organization with a view to increasing the number of patients having their inappropriate penicillin allergy de-labelled. Materials were also adapted and shared across West Yorkshire and amended for primary care. During the week, the antimicrobial pharmacy team and immunology and allergy pharmacist hosted an educational session to pharmacy teams describing the harm an inappropriate label can cause and some case studies. In the wider organization, posters, a newsletter, a ‘druggle’ and screensavers were utilized to deliver communications.

**Objectives:**

increase awareness of the harm that an inappropriate penicillin allergy label can cause; increase awareness of the penicillin allergy assessment and management guidance; encourage the de-labelling of inappropriate penicillin allergy labels; and increase knowledge of appropriate allergy documentation on any prescribing records.

**Methods:**

A survey was sent out to LTHT pharmacy teams after the week to assess the impact of the week.

**Results:**

A small number of staff responded to the survey, with 86% of people stating that their knowledge increased as a result of the week and 29% of people stated that they were now aware of the penicillin allergy assessment and management guideline. Staff had taken part in a number of activities, including taking a poster to their clinical area, improved their allergy documentation and accessed the penicillin allergy guideline. The penicillin allergy assessment and management guidance received the highest number of hits so far that month, the number of hits was 43% higher in September versus an average of the previous 6 months’ worth of hits.

**Conclusions:**

In future, we aim to build on the week and host an antimicrobial allergy week each year, with a different theme. We also aim to include more patient facing information and broaden access to the educational sessions hosted for a more diverse group of healthcare professionals.

